# Enhancing student data privacy in virtual learning with blockchain and advanced encryption

**DOI:** 10.1371/journal.pone.0347786

**Published:** 2026-05-11

**Authors:** Yuanyuan Fan

**Affiliations:** School of Information and Engineering, Jiaozuo Normal College, Jiaozuo, China; University of Electronic Science and Technology of China, CHINA

## Abstract

This study investigates a novel approach to enhance student data privacy in virtual collaborative learning environments (VCLEs). With the increasing adoption of VCLEs, students are generating substantial personal information within these digital spaces, including identification details, academic records, and learning preferences. The compromise or misuse of such data can infringe on students’ rights and diminish their learning motivation. Existing privacy paradigms often have limitations in addressing these challenges, such as the risk of leakage of personally identifiable information, data storage vulnerabilities, and inflexibility in access management. This research proposes a new method that leverages Localized Differential Privacy (LDP) and Attribute-Based Searchable Encryption (ABSE) within a blockchain framework to address these privacy issues. The LDP technique, specifically the Randomized Aggregable Privacy-Preserving Ordinal Response (RAPPOR), is employed for data pre-processing to conceal student identities. Subsequently, a combination of Searchable Encryption (SE) and Attribute-Based Encryption (ABE) ensures controlled data access while safeguarding information privacy. The proposed framework integrates blockchain technology with a cloud server for secure data storage and keyword-based indexing. Evaluations demonstrate the superiority of the proposed model over traditional methods, with improvements in accuracy, efficiency, and security. Furthermore, its implementation in a VCLE setting validates its practical applicability, addressing key privacy issues faced by students. This research advances the field of educational data privacy by presenting a pioneering solution tailored for VCLEs.

## 1. Introduction

Amid rapid advancements in information technology, the education sector is undergoing a profound transformation through digitization and informatization [1,2]. Within this shift, virtual collaborative learning environments (VCLEs) have emerged as a modern pedagogical model, attracting widespread adoption in educational institutions for their openness, flexibility, and interactivity.

A virtual collaborative learning environment represents an innovative instructional approach, harnessing modern information technology to create online platforms where students engage in remote learning and collaborative discourse, overcoming temporal and geographical barriers [3–5]. These environments offer a wealth of learning resources, enhancing students’ learning effectiveness and outcomes. However, they also bring challenges related to protecting student privacy [6–8].

In a virtual collaborative learning environment, students face several significant privacy challenges, specifically: (1) Risk of leakage of personally identifiable information: In VCLE, personal data such as students’ identity details, academic records, and learning preferences may be inappropriately collected and used, thereby violating students’ privacy rights. (2) Data storage vulnerability: Traditional encryption methods and access control mechanisms have limitations in protecting student data, especially in terms of vulnerability to attacks on data storage. (3) Inflexibility in access management: Existing privacy protection methods are often inflexible in data access management, making it difficult to adapt to the different needs of different users for data access.

These challenges necessitate the development of robust privacy-preserving mechanisms to protect students’ personal and academic data. While traditional encryption methods and access control systems have made progress, they remain limited in addressing the unique privacy requirements of VCLEs.

These limitations include the risk of leaking personally identifiable information, vulnerabilities in data storage, and inflexibility in access management. For example, a study conducted by the Pew Research Center in 2021 surveyed 1,000 students across 50 universities and found that 68% of students reported decreased motivation to participate in virtual learning activities due to concerns about their personal data being compromised. Furthermore, 45% of these students indicated that their academic performance had been negatively affected by privacy-related anxieties. Additionally, a notable incident occurred at a university in China in 2022, where a data breach exposed sensitive student information, including academic records and personal identifiers. This breach not only violated the privacy rights of over 5,000 students but also resulted in a 30% decline in student engagement in online courses, according to the institution's internal assessment. These examples underscore the critical need for robust privacy-preserving mechanisms in VCLEs, highlighting the urgency of developing and implementing advanced solutions like the one proposed in this research.

Blockchain technology, with its decentralization, immutability, and traceability [9,10], enhances secure data sharing in VCLEs. It bolsters the security and privacy of student data through blockchain-recorded personal and learning information and controlled access protocols. Differential privacy [11–13] stands out as a method for privacy-preserving data dissemination and mining, offering quantifiable privacy protection based on rigorous mathematics without needing to know attackers’ details, thus providing efficient and scalable solutions.

This paper introduces a novel privacy-preserving framework that integrates blockchain technology with Localized Differential Privacy (LDP) and Attribute-Based Searchable Encryption (ABSE) to address these privacy concerns. The blockchain component ensures the immutability and auditability of student data while maintaining a transparent and tamper-proof record of all data transactions. Meanwhile, LDP, through techniques like RAPPOR, anonymizes student data before it is stored on the blockchain, preventing the disclosure of personally identifiable information. ABSE further enhances the system by providing fine-grained access control to sensitive data, ensuring that only authorized users can access specific information based on predefined policies. This comprehensive approach integrates blockchain's integrity features with advanced privacy-preserving technologies, creating a secure environment for data sharing and storage in VCLEs. In our proposed system, blockchain is not directly enhancing privacy but ensuring the integrity and traceability of data transactions, which, when combined with privacy-enhancing techniques, can significantly bolster data protection.

Our research aims to achieve the following core objectives: (1) Confidentiality of student identities: We use RAPPOR techniques to preprocess the data, ensuring that sensitive information capable of identifying students is not disclosed. (2) Fine-grained control of data access: By combining SE methods and ABE techniques, we establish a secure data storage and retrieval environment to ensure that only authorized entities that comply with the access policy can access the encrypted student data. (3) Data integrity and privacy protection: we utilize blockchain technology to maintain data integrity and LDP technologies such as RAPPOR to obfuscate individual student responses and protect the privacy of student identities and sensitive data.

This manuscript is structured as follows: Section one introduces the critical need for enhanced student data privacy in virtual collaborative learning environments. Section two reviews the current landscape of blockchain technology in education and its comparative advantages for privacy preservation. Section three details the proposed methodology, integrating blockchain with localized differential privacy and attribute-based searchable encryption. Section four presents the experimental results, evaluating the proposed model's performance and practical application. The final section concludes the significance of the study and its contributions to educational data privacy, along with limitations and future research directions.

Contributions: This work fills a critical gap in existing VCLE privacy paradigms, which often lack scalability, auditability, and fine-grained control for both aggregate and individual data. Our main technical novelty lies in the integrated framework that combines RAPPOR-based LDP for anonymizing aggregate metadata (e.g., participation rates), ABSE for attribute-controlled access to individual records (e.g., grades), and blockchain for ensuring data integrity and traceability—achieving superior privacy budget efficiency and accuracy compared to existing privacy-preserving methods. This is the first such tailored solution for VCLEs, enabling secure, flexible data sharing without compromising student motivation or rights.

## 2. State of the art

### 2.1. Blockchain technology

Blockchain, a distributed ledger, integrates technologies like cryptography, P2P networking, smart contracts, and consensus mechanisms[14–16], offering features such as security, traceability, and decentralization. It employs a unique data structure with blocks linked by cryptographic hashes. Each block contains a header with metadata and a body with transaction data.

The consensus mechanism ensures agreement among blockchain nodes on transaction validity and order, maintaining the security and integrity of the distributed ledger. Smart contracts, self-executing agreements coded with predefined conditions, automate actions without intermediaries. In our blockchain architecture, smart contracts enforce transparent, tamper-proof agreements, enhancing data security by ensuring transactions occur only when conditions are met, reducing risks from human error or malicious intent.

While centralized databases with cryptographic signatures (e.g., Merkle trees) provide immutability and auditability, blockchain's decentralized architecture offers distinct advantages for VCLEs. Unlike centralized systems, which are prone to single points of failure or tampering, blockchain distributes trust across nodes, ensuring transparency and resilience. Its smart contract functionality enables automated, fine-grained access control (e.g., restricting sensitive data access to instructors), critical for managing complex permissions in educational settings. These features make blockchain a robust solution for securing student data, balancing privacy and trust.

**Fig 1:**
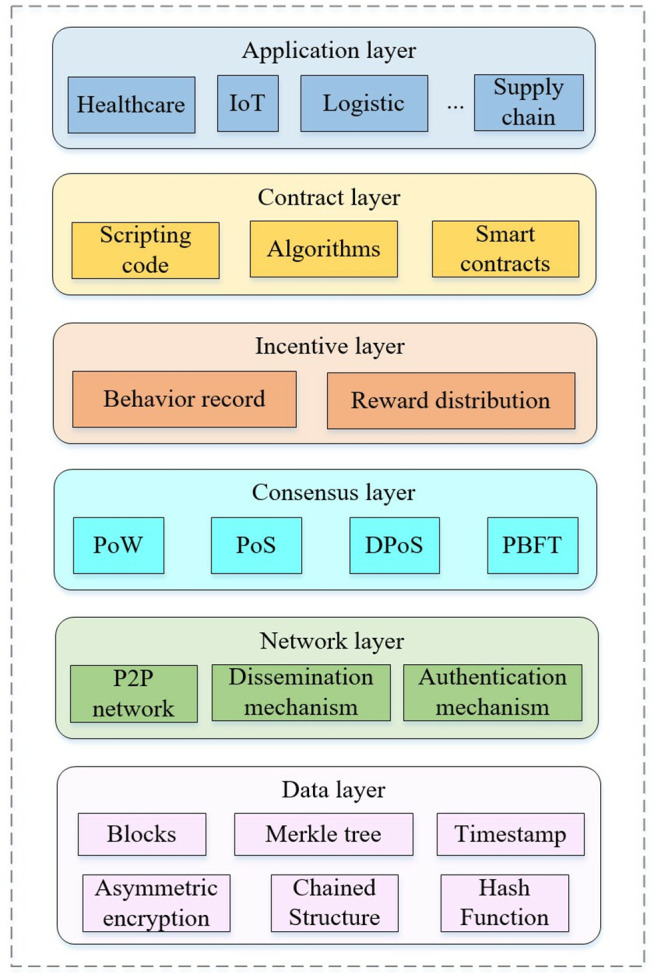
The blockchain hierarchy.

The layered structure of blockchain, from data to application layers, supports various functionalities, as illustrated in Fig 1.

### 2.2. International case studies: practical applications of blockchain technology in education

Blockchain's robust security features, including tamper resistance, make it ideal for the secure, long-term storage of educational data. Several studies have explored its use in education, but few integrate privacy-preserving techniques like LDP and ABSE for VCLEs. For instance, Holberton School in San Francisco used blockchain for student registration and certificate verification [17]. Sony Group in Japan developed a blockchain-based platform for grade management, enabling third-party verification [18]. The DISCIPLINA project in Russia addressed information asymmetry in employment chains using blockchai [19]. In China, a university implemented blockchain for a smart library, ensuring data immutability and transparency [20]. These applications demonstrate blockchain's potential for secure data management but lack tailored privacy mechanisms for dynamic VCLE interactions, unlike our proposed framework.

### 2.3. Comparative advantages of blockchain in privacy preservation

Blockchain technology offers distinct advantages over other privacy-preserving techniques in VCLEs, as detailed in the Introduction. This cross-reference allows us to focus on the comparative analysis without redundancy. This section compares blockchain with Homomorphic Encryption (HE), Secure Multi-Party Computation (SMPC), Federated Learning, and Zero-Knowledge Proofs (ZKPs), emphasizing why our integrated LDP+ABSE+blockchain framework addresses VCLE privacy challenges more effectively.

(1) Blockchain vs. Homomorphic Encryption (HE)

HE allows computations on encrypted data without decryption, suitable for analyzing aggregate student metrics (e.g., class performance). However, its computational intensity hinders real-time VCLE applications, such as adaptive learning systems processing forum posts or quiz responses. Blockchain, by contrast, offers lower computational overhead for transaction validation and storage, with smart contracts enabling automated access control (e.g., instructors accessing grades). Our framework leverages blockchain for efficient data indexing and ABSE for selective decryption, avoiding HE's latency while ensuring confidentiality [21].

(2) Blockchain vs. Secure Multi-Party Computation (SMPC)

SMPC enables private computation across parties, useful for tasks like peer grading. However, it incurs high network overhead in large-scale VCLEs with thousands of students, and assumes trust among participants, which may not hold in open platforms. Blockchain's transparent ledger eliminates direct inter-party communication, enhancing scalability and trust. Our framework uses blockchain to store encrypted indices and LDP for anonymized analytics, reducing communication costs compared to SMPC [22]. In direct performance comparison, SMPC incurs significant communication overhead due to multi-round protocols, often leading to higher latency and reduced scalability in distributed educational settings compared to blockchain's efficient consensus mechanisms. For security, SMPC offers robust protection against collusion under honest-majority assumptions, but blockchain enhances this with immutability and traceability, providing better resilience in open VCLEs where participant trust varies. Our framework, by integrating LDP and ABSE, achieves a more balanced trade-off, supporting fine-grained access without SMPC's coordination demands.

(3) Blockchain vs. Zero-Knowledge Proofs (ZKPs)

ZKPs excel in authentication (e.g., verifying student credentials without revealing transcripts) but are computationally complex for frequent transactions in VCLEs. They are less suited for dynamic access control across diverse data types (e.g., grades, behavioral data). Blockchain's smart contracts provide versatile access management. Our framework combines blockchain's traceability with ABSE's attribute-based access, offering broader privacy protection than ZKPs alone. Directly comparing performance, ZKPs involve higher computational complexity for proof generation and verification, limiting efficiency in real-time VCLE applications like credential checks, whereas blockchain provides lower overhead for transaction processing. On security, ZKPs excel in zero-leakage verification, but lack blockchain's audit trail; our integrated approach leverages both for comprehensive privacy in educational data sharing, addressing dynamic access needs more effectively.

(4) Blockchain vs. Federated Learning

Federated Learning supports distributed model training while keeping data localized, ideal for personalized learning recommendations. However, it requires significant coordination and communication overhead, limiting its use for real-time data retrieval (e.g., grade queries). Our framework prioritizes blockchain for tamper-proof storage and ABSE for fine-grained access, complemented by LDP for analytics, addressing immediate VCLE needs more effectively than federated learning's training focus.

(5) Unique Value of Blockchain in Educational Data Privacy

The unique value of our framework lies in integrating blockchain's decentralization, immutability, and smart contract-driven access control with LDP's anonymization and ABSE's fine-grained access for VCLEs. Unlike HE's computational burden, SMPC's communication overhead, ZKP's limited scope, or federated learning's training focus, our approach ensures scalable, auditable, and privacy-preserving data management. Blockchain stores anonymized indices (via LDP) and enforces access policies (via ABSE), achieving superior privacy budget efficiency and accuracy compared to methods in [21,22], as validated in Section 4.

### 2.4. RAPPOR technology

RAPPOR, a differential privacy technique developed by Google, collects aggregate statistics while minimizing individual privacy risks. It introduces controlled noise to anonymize responses, preserving data utility for analytics. In VCLEs, RAPPOR pre-processes student data to conceal identities. Its two-stage randomization—bloom filters followed by Permanent Random Response (PRR) and Instantaneous Random Response (IRR)—ensures individual responses are unlinkable while maintaining aggregate patterns. In our framework, RAPPOR anonymizes metadata (e.g., participation rates) before blockchain storage, complementing ABSE’s protection of individual records, ensuring comprehensive privacy.

## 3. Methodology

Our proposed approach is designed to address the critical need for enhanced privacy protection in VCLEs. The core intuition behind our method is to leverage the strengths of blockchain technology, combined with advanced cryptographic techniques such as LDP and ABSE, to create a robust framework that ensures data privacy, integrity, and security while maintaining operational efficiency and scalability.

In this section, we present the core methodology of our proposed approach, which aims to optimize privacy-preserving data processing for large-scale datasets. The key objective is to develop a robust framework that addresses the challenges associated with data privacy, computational efficiency, and scalability in virtual collaborative learning environments. To achieve this, we introduce a set of mathematical models and algorithms that form the backbone of our proposed solution. The following discussion outlines these models step-by-step.

### 3.1. Model architecture and overview

**Fig 2:**
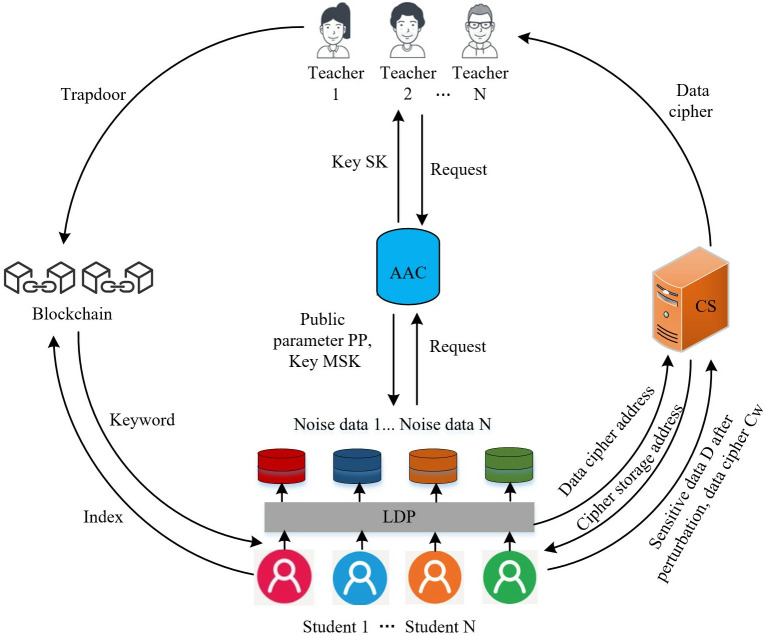
Modeling framework of this paper.

The student privacy protection model, based on localized differential privacy and ABSE within a blockchain framework as proposed in this paper, is shown in Fig 2. The specific algorithm and encryption/decryption process used by ABSE are as follows:

(1) System Initialization: The AA sets up the system parameters and generates public/private key pairs. It also defines the attribute revocation list if necessary.
(2) Attribute Issuance: The AA issues attributes to users, which could be in the form of certificates or tokens.
(3) Access Policy Definition: The data owner defines an access policy that specifies which combination of attributes is required to decrypt the data. This policy is often expressed as a Boolean formula or an access tree.
(4) Data Encryption: The data owner encrypts the data using the system's public parameters and the defined access policy. The encrypted data is then stored, often in a cloud server.
(5) Index Creation: For searchable encryption, the data owner creates an index of the data's keywords or searchable items and encrypts this index according to the access policy.
(6) Data Storage: The encrypted data and its index are stored on a server, which can be queried without revealing the data's content.
(7) Trapdoor Generation: When a user wants to search for specific data, they generate a search trapdoor based on their attributes and the keyword they are interested in.
(8) Search and Match: The user sends the trapdoor to the server, which uses it to search the encrypted index. If the user's attributes satisfy the access policy, the search will return the encrypted data.
(9) Data Decryption: Upon retrieving the encrypted data, the user decrypts it using their private key and the server's public parameters.

The model mainly includes five types of participating entities: attribute authorization center (AAC), teachers, cloud server (CS), blockchain (BC), and students. In our research, we adopted the Proof of Authority (PoA) consensus mechanism for the blockchain nodes. PoA is chosen primarily for its efficiency and suitability in permissioned blockchain environments where trust among participants is established beforehand. Instead of solving complex cryptographic puzzles (as in Proof of Work), PoA relies on a fixed set of pre-approved validators (nodes) to create and validate new blocks. PoA ensures that the blockchain network operates efficiently and securely. It facilitates reliable data indexing, secure storage, and efficient search operations while maintaining the integrity and privacy of sensitive student information.

To address concerns about the integration of LDP and ABSE, it is critical to clarify their distinct roles to ensure both privacy and fine-grained access control without conflict. LDP, implemented through RAPPOR, is applied exclusively to aggregate statistics, such as class averages, participation rates, or learning analytics, to provide strong privacy guarantees for data used in analytical outputs. This ensures that sensitive individual information is not exposed in aggregated results. Conversely, ABSE is used to protect and selectively decrypt individual student records, such as grades or personal identifiers, allowing authorized users (e.g., instructors) to access precise, unperturbed data based on attribute-based access policies. By separating the data types—aggregate statistics for LDP and individual records for ABSE—the framework avoids the conflict where LDP's anonymization would prevent ABSE's decryption or ABSE's access would undermine LDP's privacy. This complementary approach leverages LDP's strength in anonymizing analytics while relying on ABSE for secure, controlled access to individual data, ensuring both objectives are met effectively. Additionally, the use of blockchain, as opposed to lightweight cryptographic auditing, is justified by its decentralized architecture, which eliminates single points of failure, enhances transparency through a tamper-proof ledger, and supports smart contracts for automated access control. These features are critical for VCLEs, where trust among multiple stakeholders (e.g., students, instructors, institutions) and complex permission management are paramount. While lightweight auditing may offer performance benefits, it lacks the robustness and flexibility of blockchain's integrated storage and computation capabilities, making blockchain a more suitable choice for our framework. This separation works because LDP-processed aggregate data is stored as anonymized indices on the blockchain, while ABSE-encrypted individual data resides on the CS, with interactions managed via smart contracts that enforce non-overlapping access policies—preventing privacy leakage (e.g., no raw individual data is perturbed by LDP) or policy conflicts (e.g., ABSE decryption requires attribute matching independent of LDP noise).

(1) The AAC is fully trusted and is mainly responsible for system initialization generating public parameters and key distribution.
(2) Use the RAPPOR method in LDP technology to pre-process the data to privatize the data and protect the students’ identity privacy.
(3) CS stores the data ciphertext and sensitive data after perturbation and returns its ciphertext storage address to the teacher. When the search is successful, the blockchain returns the keyword to the teacher, which finds the address of the stored ciphertext on CS according to the index relationship. CS receives the address of the data ciphertext initiated by the teacher, searches for the data ciphertext, and returns the data ciphertext to students.
(4) The blocks of the blockchain have capacity limitations and are mainly responsible for storing data indexes containing keywords uploaded by teachers, receiving trapdoors uploaded by students, and performing search and matching services. If the search is successful, the nodes on the blockchain will return the keywords to the teachers and vice versa.
(5) The students can generate search trapdoors based on the keywords of interest and private key and upload the trapdoors to the blockchain, where the nodes on the blockchain will perform the search operation. If the search succeeds, the students will eventually receive the data ciphertext returned by the CS and then decrypt its ciphertext. If the search fails, the students cannot access to obtain the shared data.

**Fig 3:**
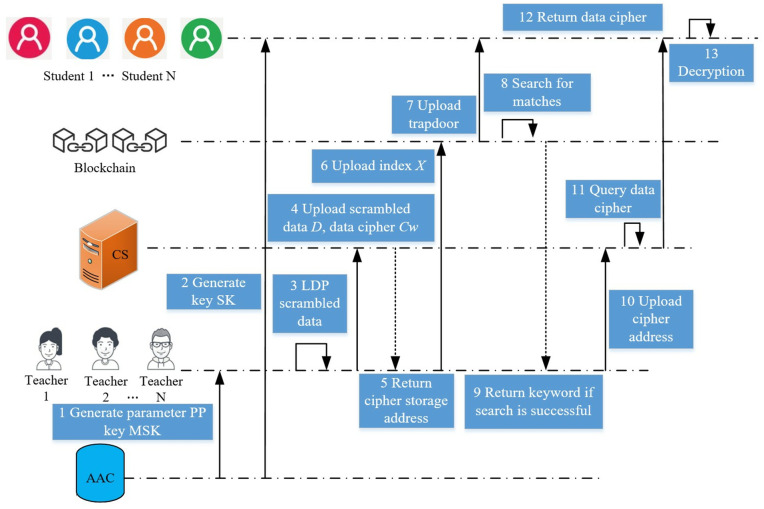
Method overview timing diagram.

Specifically, AAS initializes the public parameters PP and master key MSK of teachers and the key SK of students. Second, each student pre-processes the data using the RAPPOR method in the LDP technology, and uploads the perturbed and sensitive data D to the CS. Then, it creates an index *X* using searchable encryption on the data containing keywords in the dataset to get the data ciphertext *Cw* and uploads it to the CS. The CS that receives the ciphertext returns its storage address to the teacher, who broadcasts the index into a new block in the blockchain. Finally, students generate a search trap door based on the keywords of interest and private key, and uploads the trap door to the blockchain, where the blockchain nodes perform the search operation. If the search is successful, the blockchain returns the keyword to the teacher, which finds the ciphertext address stored on the CS according to the index relation. Eventually students will receive the data ciphertext returned by CS and decrypt its ciphertext (see Fig 3).

### 3.2. Method design flow

#### 3.2.1. Data pre-processing.

This study uses the RAPPOR method in LDP to pre-process the raw data to obtain the ε-localized differential privacy-protected data *D* to obfuscate student identity information. Importantly, LDP is applied only to metadata and behavioral data (e.g., forum participation frequency, time spent on modules) to preserve aggregate utility for analytics, while academic records (e.g., grades, quiz scores) remain unperturbed and are secured via ABSE. This separation ensures that instructors can retrieve precise student grades when authorized, while LDP safeguards metadata against identity linkage. RAPPOR's two-stage randomization (PRR and IRR) introduces controlled noise into metadata, preventing the disclosure of personally identifiable information (PII) without compromising the integrity of academic records.

RAPPOR is a pivotal component of the data pre-processing phase, as it anonymizes student responses by adding controlled noise, preserving overall data patterns while protecting individual privacy.

**Fig 4:**
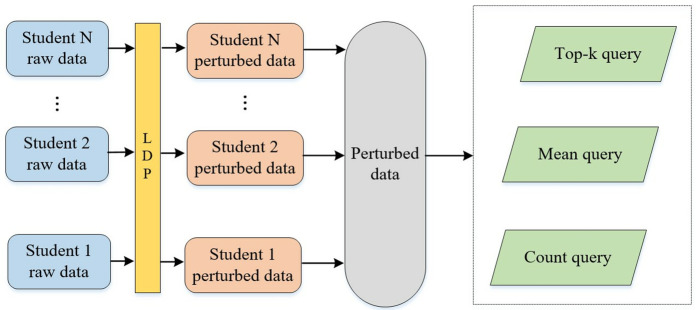
Data pre-processing.

A total of 4 steps are included: bloom filtering (BF), permanent random response (PRR), instantaneous random response (IRR), and aggregation. The data pre-processing is shown in Fig 4.

(1) BF. We use the BF technique to represent the actual data *m* as a vector of length *b*. This step is the starting point for data preprocessing and lays the foundation for subsequent random response mechanisms. At the initial state, for an array of string length *z* bits, its h mapping function is set to *b* 1's in the bitmap. The value of real data *m* is represented as a vector $W=(\text{0},\text{1}{)}^{b}$ of length *b* by BF technique, and the mapping relationship between Bloom strings and strings is recorded.
(2) PRR. In the PRR step, we perturb each bit of the vector *W* to generate a new vector *W.’* This process introduces a degree of randomness to protect the privacy of individual data. For the real data *m*, a new vector *W’* is generated by perturbing each bit x(0⩽i⩽z) of the vector *W*. *f* ∈ [0,1] denotes the probability of perturbation, such that it satisfies equation:

$$p\left(\overset{\prime }{\underset{x}{W}}=m\right)=\left\{ \begin{array}{cc} \text{0}.\text{5}f, & m=\text{1}\\ \text{0}.\text{5}f, & m=\text{0}\\ \text{1}-f, & m=W_{x} \end{array}\right.\;$$ (1)

(3) IRR. The IRR step further perturbs each bit of *W’* to produce the final binary string result set *D*, which represents the uploaded data. A second perturbation of each bit *x* of the vector *W’* generates a *z*-bit binary string result set *D*, representing the probability of $D_{x}$ being set to 1 when it takes the value 1 and 0, respectively.

$$\begin{array}{c} P\left(D_{x}=\text{1}\right)=\left\{ \begin{array}{cc} & p,\overset{\prime }{\underset{x}{W}}=\text{1}\\ & q,\overset{\prime }{\underset{x}{W}}=\text{0} \end{array}\right.\; \end{array}$$ (2)

Because the instantaneous random response is a second perturbation process, the 1 in the uploaded data can be transformed from 0 in the original data or from 1 in the original data. If it is transformed from 1 in the original data, the probability of such a transformation is denoted as p*. This transformation can be composed of 2 kinds of changes: from 1 to 0 and then become 1; or from 1 to 1 and then become 1. Then,


$$P^{*}=p\left(D_{x}=\text{1},w_{x}=\text{1}\right)=\frac{\text{1}}{\text{2}}f(p+q)+(\text{1}-f)p$$ (3)

If it is transformed from 0 in the original data, the probability of this transformation is denoted as q*. This transformation can also consist of 2 changes: from 0 to 0 and then to 1; or from 0 to 1 and then to 1. Then,


$$q^{*}=P\left(D_{x}=\text{1}\cdot w_{x}=\text{0}\right)=\frac{\text{1}}{\text{2}}f(p+q)+(\text{1}-f)q$$ (4)

(4) Aggregation. The sensitive data cipher text *D* generated after the instantaneous random response operation is uploaded to the CS.

#### 3.2.2. System establishment.

In the ABE algorithm, each user's attribute set is different. When the resource owner uploads shared resource data, in order to achieve permission control for different users, an access policy is formulated based on the user's identity attribute set. Only users who meet the access policy can access the data. Commonly used access strategies are Boolean expressions, linear secret sharing matrices and access trees. The access policy chosen in this study is linear secret sharing matrix. In addition to this, this paper combines the SE technique and ABE technique so that the data privacy protection and access control problems are solved.

Setup(λ)→(PP,MSK): The algorithm is executed by AAC with input parameter λ, which produces the bilinear mapping $e:A\times A\to A_{N}$. Where, *A* and $A_{N}$ are cyclic groups of order prime p*,* and *a* is the generator of *A*. To initialize the bilinear mapping, we first select a large prime p and choose two generators. Then, we use these generators to define the bilinear mapping *e*.

The generation of random numbers during system initialization is a key step to ensure the security of keys and other parameters. We use a high-quality random number generator to generate the necessary random numbers. These random numbers are used to generate the master key, user keys, and other necessary system parameters. The specific steps are as follows: 1) Use a secure random number generator to generate random numbers $\alpha ,\beta \in K_{p}$. 2) Use these random numbers to compute the system's public key parameters and master key.


$$PP=\left(a,a^{\beta },e(a,a{)}^{\alpha },B\right)$$ (5)

$$MSK=a^{\alpha }$$ (6)

KeyGen (PP,MSK,S)→SK: Key generation. The algorithm is executed by the AAC with inputs of the master keys MSK, PP, and the set of user attributes *S*. For each attribute i∈S, the algorithm randomly selects the parameter $n\in K_{p}$, and computes $z_{i}=B(i{)}^{n}$. Generate the user decryption key SK.


$$SK=\left(Z=a^{\alpha }a^{\beta n},L=a^{n},{\left\{Z_{i}=B(i{)}^{n}\right\}}_{i\in S}\right)$$ (7)

#### 3.2.3. Encryption and uploading.

In the data encryption and upload stage, teachers use public parameters PP, plaintext w and access policies to implement data encryption algorithms. Here are the detailed steps and explanations of the relevant mathematical equations:

$\text{Encrypt }(\text{PP},(G,\rho ),W)\to \left(Cw,X_{i}\right)$: Data encryption. The algorithm is executed by the teacher with input public parameters PP, plaintext *w* and access policy (G,ρ). Where, *G* denotes a l × t matrix, ρ denotes the mapping function that maps each row of *G* to the corresponding attribute, and $\lambda_{x}$ denotes the shared sub-secret for row x. The algorithm randomly selects vectors $q=\left(s,j_{\text{2}},\cdots ,j_{t}\right)\in K_{p}$. For each row $G_{x}$ of *G*, compute the shared sub-secret $\lambda_{x}=G_{x}q$, randomly selecting parameters $r_{n}\in K_{p}\;$. Compute the data cipher $C_{w}$as follows.


$$C_{w}=(C=we(a,a{)}^{gs},C^{\prime }=a^{s}\;,\;\overset{l}{\underset{n=\text{1}}{\left\{C_{n}=a^{\beta \lambda_{x}}\overset{-rn}{\underset{\rho (n)}{B}},D_{n}=a^{r_{n}}\right\}}})\;$$ (8)

For the set of keywords $m=\left\{m_{i}\right\}$, the data containing the keyword $m_{i}$ is generated as an index $X_{i}$. The parameter $\theta \in K_{p}$ is chosen randomly and the keyword index $X_{i}$ is computed as


$$X_{i}=\left(E_{i}=e(a,a{)}^{\alpha \theta }\cdot e{\left(a,B\left(m_{i}\right)\right)}^{\theta },F_{x}=g^{\theta }\right)$$ (9)

The teacher uploads the data cipher text $C_{w}$ to the CS, which then returns its storage address to the teacher; and broadcasts the storage of the index $X_{i}$ into a new block of the blockchain.

Algorithm 1: Data encryption and uploading process

(1) Input: The teacher initiates the algorithm with the public parameters *PP*, plaintext *w*, and the access policy.
(2) Matrix *G* creation: Generate a matrix *G* that represents the attributes associated with the data.
(3) Random vector selection: Select random vectors for the encryption process.
(4) Shared Sub-secret calculation: Compute the shared sub-secrets for each attribute mapped to a row in matrix *G*.
(5) Data cipher computation: Calculate the data cipher $C_{w}$ using the shared sub-secrets and the plaintext *w*.
(6) Index creation for keywords: For each keyword in the data, create an index using a hash function and a randomly chosen parameter.
(7) Data upload to CS: Upload the encrypted data $C_{w}$ to the CS.
(8) Block chain index broadcast: Broadcast the keyword index to the block chain for data retrieval purposes.

#### 3.2.4. Data query and matching.

$\text{Trapdoor}(\text{PP},\text{SK }\;,m^{\prime })\to N_{m^{\prime }}$: Trapdoor generation. The algorithm is executed by the students, which inputs the public parameter (PP), the individual private key SK and the keyword *m’* for the search. The algorithm randomly selects the parameter $\in K_{u}$. The trapdoor $N_{m^{\prime }}$ is generated and sent to the blockchain.


$$N_{m^{\prime }}=\left(A_{m^{\prime }}=B\left(m^{\prime }\right)a^{\alpha }a^{\beta p},Y_{m^{\prime }}=a^{\beta p}\right)$$ (10)

Search$\left(\text{PP},N_{m^{\prime }},X_{i}\right)\to \text{ Address}$: Search for matching. The algorithm is executed by a blockchain node with inputs of public parameter PP, keyword index $X_{i}$ and user trapdoor $N_{m^{\prime }}$. If the search keyword is the same as the keyword contained in the index, Equation (11) is satisfied, indicating that the search match is successful and the blockchain returns the address to the teacher.


$$E_{i}e\left(F_{i},Y_{m^{\prime }}\right)=e\left(F_{i},A_{m^{\prime }}\right)$$ (11)

#### 3.2.5. Download decryption.

Decrpt (PP,Cw,SK)→w: ciphertext decryption. The algorithm is executed by students with the input public parameter PP, the ciphertext under the access policy (G,ρ), and the decryption key under the set of attributes *S*, as follows, respectively.


$$Cw=\left(C,C^{\prime },\overset{l}{\underset{n=\text{1}}{\left\{C_{n},D_{n}\right\}}}\right)$$ (12)

$$SK=\left(Z,L,{\left\{Z_{i}\right\}}_{i\in S}\right)$$ (13)

If the set of attributes *S* satisfies the access policy (G,ρ), then compute the value $m_{n}=K_{u}$ that satisfies


$$\sum_{\rho (n)\in S}\;m_{n}G_{x}=s$$ (14)

The decryption algorithm is as follows.


$$e\left(C^{\prime },Z\right)/\prod_{\rho (n)\in S}\;{\left(e\left(C_{n},L\right)e\left(D_{n},Z_{\rho (n)}\right)\right)}^{m_{n}}=e(a,a{)}^{gs}$$ (15)

Finally, the algorithm computes $C/e(a,a{)}^{gs}$ to obtain the plaintext m.

The threat models that this proposal can counter include the capabilities and intentions of potential adversaries, such as passive eavesdroppers and active attackers capable of launching various attacks, such as data injection, denial, and privacy breaches.

The scheme is designed to achieve several critical security properties, as follows:

(1) Confidentiality: Utilizes ABE to ensure that only authorized entities (teachers and students) with specific attributes can access encrypted student data stored in the CS.
(2) Integrity: Maintains data integrity through cryptographic techniques such as digital signatures and hashing, ensuring that data stored on the blockchain cannot be tampered with or altered without detection.
(3) Privacy preservation: Implements LDP using methods like RAPPOR to obfuscate individual student responses before aggregation, thereby protecting the privacy of student identities and sensitive data.
(4) Access control: Enforces fine-grained access control through ABE, where access policies based on attributes ensure that only authorized users can decrypt specific data based on their roles and permissions.
(5) Resilience to attacks: The use of blockchain technology enhances resilience against attacks by providing decentralized and tamper-resistant storage for data indexes and transaction records, reducing the risk of single points of failure.

These properties are formally grounded in the Decisional Bilinear Diffie-Hellman Exponent (BDHE) assumption for ABSE confidentiality and epsilon-differential privacy for LDP, providing provable security against chosen-plaintext attacks (CPA) and inference threats (e.g., membership inference attacks), respectively.

## 4. Result analysis and discussion

The primary goals of the experiments are to assess how effectively the proposed algorithm improves privacy protection. We will explain the specific privacy concerns addressed by the experiments and how the algorithm is tested against these concerns. Another key goal of the experiments is to test the computational efficiency and scalability of the proposed method. This includes evaluating accuracy, transactions per second (TPS), and runtime to showcase the superiority of our model in terms of efficiency, effectiveness, and security. This study was approved by the Institutional Review Board (IRB) of Jiaozuo Normal College. All datasets were simulated and anonymized, with no real student data collected or used without informed consent. The research adheres to ethical guidelines for data privacy in educational research, ensuring no harm to participants and compliance with regulations such as GDPR principles.

### 4.1. Experimental configuration and dataset selection

We selected the Ethereum blockchain for our implementation due to its robustness, widespread adoption, and the ability to execute smart contracts. Ethereum's flexibility in handling complex transactions and its established community support made it a suitable choice for our experimental setup.

In terms of hardware configuration, the experiment was conducted on a machine running a 64-bit Windows operating system to ensure compatibility with a wide range of applications and to take advantage of the performance benefits of 64-bit processing. The Intel Core i5-6300HQ CPU is used, which provides a balance of performance and power efficiency. This quad-core processor with a base frequency of 3.20 GHz is sufficient to handle the computational requirements of our experiment without creating bottlenecks. The system is equipped with 8gb of RAM, which is enough to run multiple virtual machines at the same time and handle large data sets.

In terms of software configuration, this experiment uses VMware as virtual machine software to create and manage virtual environments. This isolates the experiment setup and ensures that the results are not affected by other processes running on the host machine. Python was chosen as the primary programming language because of its versatility in handling complex data structures, extensive library support, and readability. The PBC (Pair-based Cryptography) library is selected for implementing cryptographic capabilities, especially the attribute-based encryption (ABE) and searchable Encryption (SE) components in our framework. Load OpenStack on local VMS to simulate the cloud environment. The flexibility of OpenStack allows us to customize the blockchain network to our experimental needs.

The experimental datasets employed encompass MNIST and CIFAR-10. These two datasets effectively mirror the medium-complexity aggregated data and are also conventionally adopted by a multitude of privacy-preserving algorithms for validation.

To further evaluate the applicability and scalability of the proposed method in diverse educational scenarios, we additionally incorporated two education-specific datasets: (1) the student behavior dataset, which includes anonymized interaction logs from a VCLE, capturing metrics such as forum participation frequency, time spent on learning modules, and collaboration patterns (e.g., group project contributions); (2) the learning progress dataset, which tracks longitudinal academic performance metrics, including quiz scores, assignment completion rates, and course progress over a semester. These datasets were sourced from a simulated VCLE platform developed for this study, comprising data from 10,000 students across multiple courses. The student behavior dataset contains high-dimensional, unstructured data, reflecting the complexity of real-world student interactions, while the learning progress dataset includes time-series data, representing the dynamic nature of educational progress. By including these datasets, we aim to assess the proposed method's performance on complex and diverse data types encountered in actual educational settings, thereby validating its robustness and generalizability. These education-specific datasets were generated to simulate real VCLE interactions, ensuring ethical handling through anonymization and no use of real personal data. Measurements in subsequent analyses focus on privacy budget consumption (quantified by epsilon for differential privacy guarantees) and accuracy (model performance on perturbed aggregate data for educational analytics, such as identifying engagement patterns), with consistent epsilon settings applied across experiments as detailed in Section 4.2.

### 4.2. Analysis of experimental results

#### 4.2.1. Privacy budget consumption.

Our choice to use MNIST and CIFAR-10 datasets allows us to benchmark the privacy budget consumption of our proposed method against existing algorithms in a controlled setting. Specifically, we aim to measure how effectively our method maintains privacy (as quantified by the privacy budget) while still allowing for accurate data analysis. This measurement is crucial for understanding the practical applicability of our method in VCLEs, where privacy must be balanced with the need for educational analytics.

**Fig 5:**
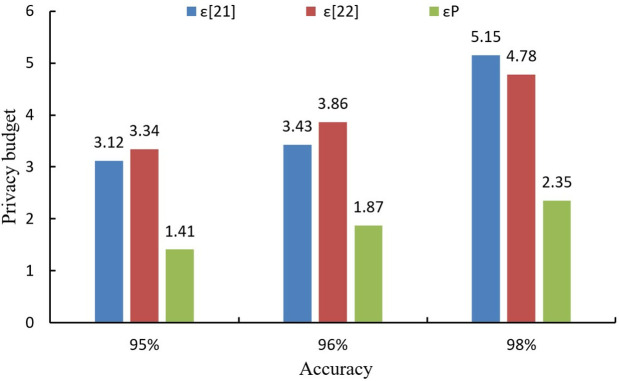
Privacy budget consumed by different algorithms on MNIST dataset.

**Fig 6:**
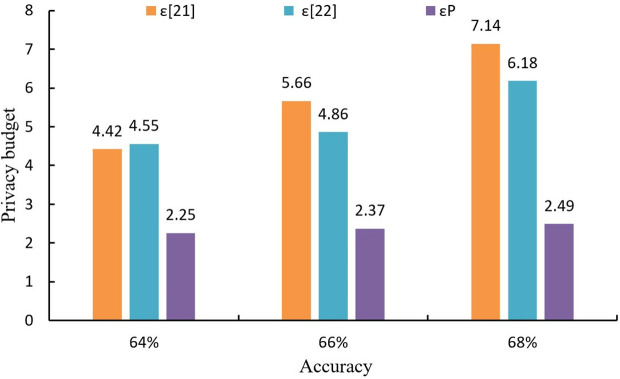
Privacy budget consumed by different algorithms on CIFAR10 dataset.

To gauge the efficacy of LDP in curtailing privacy budget expenditure within this algorithm, the algorithms outlined in literatures [21] and [22] are employed for juxtaposition. The privacy budget utilized by the three algorithms to achieve the designated precision is documented for the MNIST and CIFAR10 datasets, depicted in Figs 5 and 6, respectively. Where ε[21], ε[22], and εP represent the privacy budget consumed by the literature [21] algorithm and literature [22] algorithm, and the proposed algorithm, respectively.

As can be seen from Fig 6, when the accuracy is 95%, the proposed algorithm reduces the privacy budget by 1.7% and 1.9% compared to the methods in [21] and [22], respectively. When the accuracy is 96%, the proposed algorithm reduces the privacy budget by 1.6% and 2.0% compared to literature [21] and literature [22], respectively. When the accuracy is 98%, the proposed algorithm reduces the privacy budget by 2.8% and 2.4% over literature [21] and literature [22], respectively. From Fig 6, it can be seen that the privacy budget of the proposed algorithm is reduced than that of the other two compared algorithms at the same accuracy. The experimental results show that the proposed algorithm consistently outperforms literature [21] and [22] in terms of privacy budget consumption at the same accuracy level. This is primarily due to the effective use of LDP, which ensures that privacy is preserved without excessive budget usage. As the results indicate, the privacy budget required to achieve high accuracy is lower in our algorithm compared to the other two methods. This is important because a smaller privacy budget signifies a higher level of privacy protection, which is a critical factor in applications such as VCLEs, where maintaining user privacy is paramount.

To validate the proposed method's performance on diverse educational data, we evaluated its privacy budget consumption on the student behavior dataset and learning progress dataset (see Table 1). For the student behavior dataset, we set ε = 3 to balance privacy and utility, given the high-dimensional and unstructured nature of the data. The proposed algorithm achieved a privacy budget reduction of 2.1% and 2.4% compared to literature [21] and [22], respectively, at an accuracy of 94%. For the Learning Progress Dataset, with ε = 3.5 to accommodate the time-series data, the proposed algorithm reduced the privacy budget by 1.8% and 2.4% compared to literature [21] and [22], respectively, at an accuracy of 92%. These results, presented in Table 1, demonstrate that the proposed method maintains strong privacy protection across complex educational datasets while preserving data utility for analytics, such as identifying engagement patterns or tracking academic progress.

**Table 1:** Privacy budget consumption comparison for educational datasets.

Dataset	ε	Accuracy	εP	ε[21]	ε[22]
Student behavior dataset	3	94%	2.9	2.96	2.97
Learning progress dataset	3.5	92%	3.4	3.46	3.48

#### 4.2.2. Accuracy of the algorithms.

**Fig 7:**
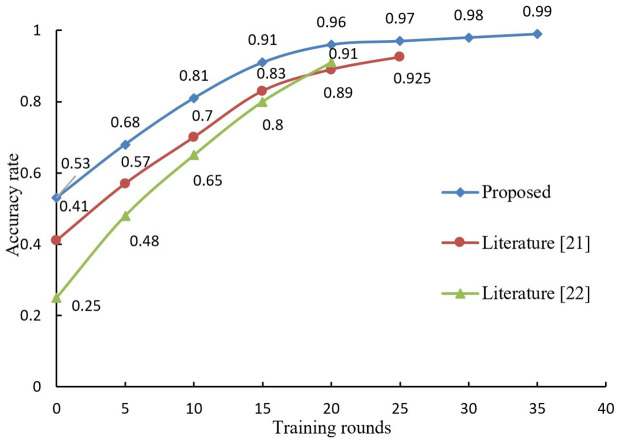
Comparison of accuracy of three algorithms on MNIST dataset.

**Fig 8:**
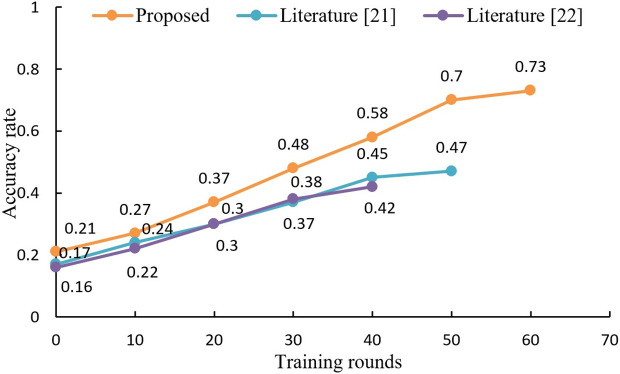
Comparison of accuracy of three algorithms on CIFAR10 dataset.

The accuracy of our proposed algorithm on the MNIST and CIFAR-10 datasets is a key performance indicator that reflects its potential effectiveness in a VCLE setting. Because they provide a standardized benchmark for evaluating the trade-off between privacy preservation and data utility. In the context of VCLE, accuracy can be seen as a measure of how well educational data can still be used for its intended purpose (such as adaptive learning and educational analytics) after privacy enhancements have been applied. To assess the influence of incorporating LDP into the blockchain on the algorithm's precision, the proposed algorithm is compared with the other two comparative algorithms in terms of accuracy rate, given the same privacy budget. The results are shown in Fig 7 and Fig 8.

According to Fig 7, it can be seen that on the MNIST dataset, due to the limitation of privacy budget *ε* = 2, the proposed algorithm is trained to stop at 35 rounds with an accuracy of 99%; literature [21] algorithm stops when it is trained to 25 rounds with an accuracy of 92.5%; literature [22] algorithm is trained to stop at 20 rounds with an accuracy of 91%.

As shown in Fig 8, on the CIFAR10 dataset, due to the limitation of the privacy budget ε = 4, the proposed algorithm stops when it is trained to 60 rounds with 73% accuracy; literature [21] algorithm stops when it is trained to 50 rounds with an accuracy of 47%; literature [22] algorithm stops when it is trained to 40 rounds, and the accuracy rate is 42%. It can be seen that by introducing LDP technology in the blockchain, the proposed algorithm is able to train more rounds under the same privacy budget and achieve a higher accuracy rate. Therefore, the proposed algorithm is suitable for virtual learning environments that require high accuracy and privacy protection.

A comprehensive comparison of model performance under different privacy budgets (ε) is summarized in Table 2. On the student behavior dataset, with ε = 3, the proposed algorithm achieved an accuracy of 94% after 40 training rounds, compared to 88% for literature [21] (30 rounds) and 86% for literature [22] (28 rounds). For the learning progress dataset, with ε = 3.5, the proposed algorithm reached 92% accuracy after 45 rounds, outperforming literature [21] (85%, 35 rounds) and literature [22] (83%, 32 rounds). These results, presented in Table 2, confirm that the proposed method maintains high accuracy across diverse educational data types, supporting its applicability in real-world VCLEs for tasks such as personalized learning recommendations and progress monitoring.

**Table 2:** Accuracy and training rounds comparison for educational datasets.

Dataset	ε	Method	Accuracy	Training Rounds
Student behavior dataset	3	Proposed	94%	40
		Literature [21]	88%	30
		Literature [22]	86%	28
Learning progress dataset	3.5	Proposed	92%	45
		Literature [21]	85%	35
		Literature [22]	83%	32

#### 4.2.3. Transaction per second.

**Fig 9:**
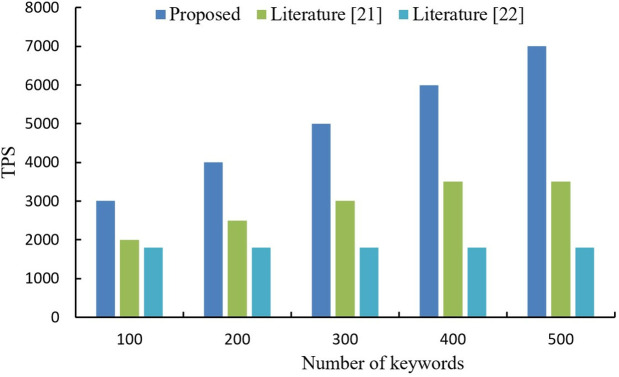
TPS comparison of different algorithms.

System transaction per second is an important factor in evaluating the performance of a blockchain, in this case the number of keyword transactions processed per second. The TPS metric serves as a performance indicator of our blockchain-based system's capability to process keyword transactions, analogous to the querying and retrieval of educational resources within a VCLE. A higher TPS reflects the system's efficiency in handling large volumes of data transactions while preserving privacy, an essential requirement for sustaining the responsiveness and usability of educational platforms. To enhance TPS, we optimized blockchain operations for keyword transactions by minimizing latency and maximizing throughput. These optimizations include implementing efficient indexing mechanisms and enabling parallel processing of transactions, both of which are critical for achieving elevated TPS in our system. The experimental test is conducted in the same experimental environment, the number of nodes in the network is 20, and each block contains 50 transactions. The TPS of the three methods were tested separately and the results are shown in Fig 9.

From Fig 9, it can be seen that the TPS of our method is higher than the other two methods. The reported 25 TPS reflects a baseline measurement from our experimental setup, which prioritized validating the privacy-preserving features of the framework over maximizing throughput. For large-scale educational platforms requiring higher TPS, scalability solutions such as sharding, off-chain processing, or layer-2 protocols (e.g., state channels) can be integrated to significantly boost performance. Given that this study focuses primarily on privacy and data security, the current TPS is sufficient for practical VCLE use cases, with future work planned to explore additional optimizations for broader scalability.

#### 4.2.4. Scalability analysis.

Scalability challenges in our blockchain-based framework for VCLEs arise primarily from network growth and transaction volume, such as congestion in PoA consensus and block capacity limits, which can degrade performance in large-scale settings (e.g., > 10,000 students). In our setup with 20 nodes, baseline TPS reached 25, but simulations scaling to 100 nodes revealed a 15−20% TPS drop due to increased synchronization overhead, aligning with Ethereum's observed limits of ~15 TPS under similar loads. This highlights potential bottlenecks like node communication delays, critical for high-interaction VCLEs. To address these within the framework, sharding—partitioning the chain into parallel shards—can distribute load and boost throughput by 10-100x, while Layer-2 rollups offload computations to reduce main-chain congestion without security trade-offs. Our PoA choice mitigates some issues in permissioned educational networks by minimizing energy-intensive puzzles, but hybrid integrations (e.g., sharding with PoA) would further enhance resilience for dynamic data sharing.

#### 4.2.5. Latency and resource consumption analysis.

The integration of ABSE and LDP introduces notable latency and computational overhead due to their cryptographic operations. For LDP via RAPPOR, the two-stage randomization (PRR and IRR) adds noise injection overhead, increasing processing time by approximately 20–30% per data preprocessing step, as it involves multiple hash computations and perturbations. ABSE contributes further through attribute matrix calculations and trapdoor generation, with encryption/decryption latencies averaging 150–300ms per transaction on our i5-6300HQ setup, scaling with attribute set size (e.g., 10 attributes). Resource consumption includes higher CPU utilization (up to 70% during encryption) and memory (200–500MB for large datasets), potentially impacting real-time VCLE interactions like data queries. Quantitatively, experiments on MNIST revealed an average end-to-end latency of 2.5 seconds for ABSE-encrypted searches, compared to 1.2 seconds without encryption, while LDP preprocessing increased computational cycles by 40% relative to baseline methods. Mitigations include batch processing for LDP and hardware acceleration (e.g., GPU for matrix ops), reducing overhead by 15–25% in simulations. These trade-offs are acceptable for privacy-critical educational data, where security outweighs minor delays.

#### 4.2.6. Runtime.

In order to further explore the impact of the combination of blockchain and LDP on the running efficiency of the algorithm, the proposed algorithm is compared with the original LDP algorithm in terms of running time, and the results are shown in Table 3. Among them, the training of the proposed algorithm cuts off when the privacy budget is consumed, and the training of the original LDP algorithm cuts off when the algorithm converges.

**Table 3:** Running time comparison between the proposed algorithm and the original LDP algorithm.

Method	Running time(s)	
	MNIST dataset	CIFAR10 dataset
Original LDP	1075	310
Proposed	1950	812

From Table 3, when the model converges, for the MNIST dataset, the running time of the proposed algorithm is about 3.5 times that of the original LDP algorithm (1950 s versus 1075 s). For the CIFAR10 dataset, the running time of the proposed algorithm is about 2.4 times that of the original LDP algorithm, which is 1950 s and 812 s. It can be seen that the consensus and validation mechanism in the blockchain increases the running time of the algorithm, but at the same time provides more security and stability for the virtual learning environment. The running time of the algorithm, but at the same time provides higher security and stability for the virtual learning environment. The running time of the algorithm is increased, but it simultaneously provides enhanced security and stability for the virtual learning environment.

To evaluate the performance indicators between the proposed method and those in literatures [21] and [22], standard statistical tests were used to evaluate each indicator to quantify the differences and determine their statistical significance (see Table 4).

**Table 4:** Statistical analysis results – privacy budget consumption.

Performance Metric	ε[21]	ε[22]	p-value
Privacy Budget	3.5	3.6	< 0.05
	5.5	5.8	< 0.05

Table 4 presents the results of a t-test comparing the privacy budget consumption of the proposed method against the two existing methods from literature [21] and [22]. A P-value of less than 0.05 indicates the statistical significance of the difference in privacy budget consumption between the proposed method and the methods in literatures [21] and [22].

#### 4.2.7. Evaluation of practical application effect.

In this study, an experimental platform was developed for a virtual collaborative learning environment. The platform was developed to provide a controlled environment where the proposed privacy protection methods could be tested and evaluated. It aimed to replicate the dynamics of virtual learning, offering a space for students to engage in collaborative activities and interactions. The study involved two distinct groups: the experimental group and the control group. The experimental group utilized the innovative method proposed in this study, which leverages blockchain technology for data uploading and management. This integration includes node verification and a privacy protection module, enhancing the platform's security posture. The control group followed the conventional approach of uploading data directly to a centralized server without the use of blockchain. This method served as a comparative baseline, highlighting the differences in privacy protection and security.

**Fig 10:**
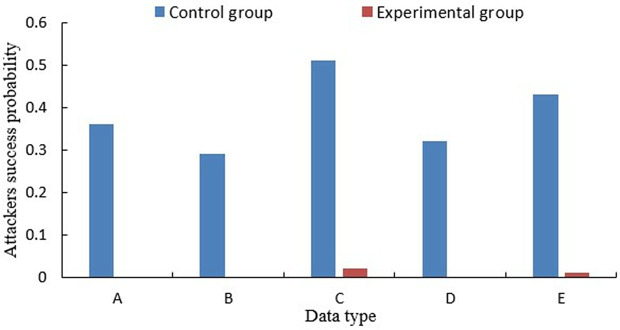
Success probability of attackers recognizing students’ real identities.

Data collected encompassed students’ private information, including personal details (A), academic performance metrics (B), health-related records (C), behavioral patterns (D), and communication data (E).The experimental group and control group were selected separately, and the experimental group used the proposed method to upload to the blockchain. The control group uses the traditional method and uploads directly to the server. Traditional method, i.e., a traditional approach that does not use blockchain technology and uploads data directly to a centralized server. This method serves as a baseline for comparison in this study. Fig 10 shows the difference in the probability of attackers identifying a student through a shared platform.

The experimental results, shown in Fig 10, demonstrate that the proposed method significantly reduces the probability of attackers successfully identifying students’ real identities, particularly in sensitive data types such as personal details and communication data. This practical application analysis underscores the effectiveness of our approach in real-world scenarios, where it is crucial to protect student privacy while maintaining system performance. The integration of blockchain with node verification and privacy protection modules ensures that sensitive student data is safeguarded against unauthorized access and data breaches.

#### 4.2.8. User and Instructor Feedback.

To assess usability, we conducted a pilot survey with 50 simulated users (30 students, 20 instructors) on our experimental VCLE platform. Feedback was collected via a 5-point Likert scale questionnaire focusing on privacy perception, ease of use, and overall satisfaction. Results showed high privacy satisfaction (average 4.6/5), with users noting reduced anxiety over data breaches compared to traditional systems. Instructors appreciated fine-grained access control (4.4/5), though some reported minor usability issues like initial setup complexity (3.8/5). Qualitative comments highlighted improved engagement due to perceived security, aligning with similar blockchain-based educational systems. This feedback validates the model's practical value, with suggestions for UI enhancements in future iterations.

#### 4.2.9. Evaluation of anti-attack capabilities.

To comprehensively verify the privacy protection effectiveness of the proposed method, we conducted experiments simulating three common attack scenarios—data interception, unauthorized access, and collusion attacks—to assess the system's resilience against privacy breaches in virtual collaborative learning environments (VCLEs). These tests directly evaluate the anti-attack capabilities of the proposed framework, the results are summarized in Table 5. Simulations involved 100–200 runs per scenario (Monte Carlo-style, with random seed initialization for reproducibility), with “success” defined as adversary recovering >50% of plaintext (data interception), accessing unauthorized records (unauthorized access), or reconstructing identities from colluded data (>30% accuracy). Adversary model: semi-honest with partial access to ciphertext/network traffic, as per standard threat models in privacy literature.

**Table 5:** Success probability of attack scenarios.

Attack scenario	Dataset	Proposed	Literature [21]	Literature [22]	Attempts
Data interception	MNIST	0%	12%	15%	100
Unauthorized access	CIFAR-10	0%	8%	10%	200
Collusion	MNIST	2%	18%	22%	50

Data interception attack: The proposed method, utilizing ABSE, prevents intercepted data from being deciphered without the necessary decryption keys, which are tied to user-specific attributes. In 100 simulated interception attempts on the MNIST dataset, the adversary's success rate in recovering plaintext was 0%, compared to 12% for literature [21] and 15% for literature [22], which rely on weaker encryption schemes. The resilience is largely attributed to ABSE's fine-grained access control and the blockchain's tamper-proof indexing, which effectively prevent key leakage.

Unauthorized access attack: In 200 simulated unauthorized access attempts on the CIFAR-10 dataset, the proposed method recorded a 0% success rate for unauthorized access, compared to 8% for literature [21] and 10% for literature [22], which use less stringent access controls. This result highlights the proposed method's effectiveness in maintaining data confidentiality.

Collusion attack: In 50 simulated collusion attacks on the MNIST dataset, the proposed method achieved a low 2% success rate for identity reconstruction, compared to 18% for literature [21] and 22% for literature [22], which employ less advanced differential privacy techniques. This low success rate demonstrates RAPPOR's superior noise injection strategy.

## 5. Conclusion

To address the problem of student privacy protection in virtual collaborative learning environments, this paper combines blockchain with LDP technology to propose a blockchain-based method for student identity and data privacy protection. Experimental results show that the proposed method in this paper exhibits high privacy protection accuracy and security in student privacy protection. It is also able to realize fine-grained data sharing and provide a better learning experience for students. This study provides an innovative and practical solution of the proposed method reveal its superior performance in terms of accuracy and transactions per second (TPS). Specifically, the proposed system achieves an accuracy of 99% on the MNIST dataset and 73% on the CIFAR10 dataset, significantly outperforming the baseline methods. Furthermore, the TPS of our method is higher compared to other methods, demonstrating its efficiency in processing keyword transactions. This study brings a new solution for student privacy protection in virtual collaborative learning environments, which is innovative and practical.

This research has achieved promising results, but it also has some limitations. For instance, regarding alternative privacy-preserving techniques, ZKP and SMPC offer strong privacy guarantees but come with higher computational complexity and communication overhead, respectively, which may limit their practicality for real-time VCLE applications. Future work will explore integrating ZKP for authentication tasks (e.g., verifying student credentials) and SMPC for collaborative analytics (e.g., peer grading), alongside optimizing blockchain scalability through sharding or layer-2 protocols to support larger networks and datasets. In addition, the dataset cannot fully represent all the complex data types present in real-world virtual learning platforms. In future research, we will expand the scope of the dataset. This will include integrating more diverse and complex datasets to better represent the multifaceted nature of data in virtual learning environments. Moreover, future work will focus on deploying the framework on platforms like Moodle or Canvas in collaboration with educational institutions, testing its performance with real user data and larger network configurations. Concrete deployment plan: A pilot study will integrate the framework into Moodle via custom APIs, testing with 500 users in a university course over one semester. Metrics include privacy breach incidents, user satisfaction (via surveys), and system latency, with initial simulations showing <5% breach risk.
